# Molecular Dynamics Simulation Combined with Neural Relationship Inference and Markov Model to Reveal the Relationship between Conformational Regulation and Bioluminescence Properties of *Gaussia* Luciferase

**DOI:** 10.3390/molecules29174029

**Published:** 2024-08-26

**Authors:** Xiaotang Yang, Ruoyu Zhang, Weiwei Han, Lu Han

**Affiliations:** Key Laboratory for Molecular Enzymology and Engineering of Ministry of Education, School of Life Science, Jilin University, 2699 Qianjin Street, Changchun 130012, China; yangxt22@mails.jlu.edu.cn (X.Y.); zhangry1321@mails.jlu.edu.cn (R.Z.); weiweihan@jlu.edu.cn (W.H.)

**Keywords:** *Gaussia* luciferase (Gluc), bioluminescence, molecular dynamics simulations, Markov model, conformational changes, neural relationship inference

## Abstract

*Gaussia* luciferase (Gluc) is currently known as the smallest naturally secreted luciferase. Due to its small molecular size, high sensitivity, short half-life, and high secretion efficiency, it has become an ideal reporter gene and is widely used in monitoring promoter activity, studying protein-protein interactions, protein localization, high-throughput drug screening, and real-time monitoring of tumor occurrence and development. Although studies have shown that different Gluc mutations exhibit different bioluminescent properties, their mechanisms have not been further investigated. The purpose of this study is to reveal the relationship between the conformational changes of Gluc mutants and their bioluminescent properties through molecular dynamics simulation combined with neural relationship inference (NRI) and Markov models. Our results indicate that, after binding to the luciferin coelenterazine (CTZ), the α-helices of the 109–119 residues of the Gluc Mutant2 (GlucM2, the flash-type mutant) are partially unraveled, while the α-helices of the same part of the Gluc Mutant1 (GlucM1, the glow-type mutant) are clearly formed. The results of Markov flux analysis indicate that the conformational differences between glow-type and flash-type mutants when combined with luciferin substrate CTZ mainly involve the helicity change of α7. The most representative conformation and active pocket distance analysis indicate that compared to the flash-type mutant GlucM2, the glow-type mutant GlucM1 has a higher degree of active site closure and tighter binding. In summary, we provide a theoretical basis for exploring the relationship between the conformational changes of Gluc mutants and their bioluminescent properties, which can serve as a reference for the modification and evolution of luciferases.

## 1. Introduction

Reporter genes play a pivotal role in studying various biological processes, including gene regulation, gene mutation analysis, and DNA hybridization analysis [1,2,3]. These genes are broadly categorized into fluorescent proteins, such as Green Fluorescent Protein (GFP), and bioluminescent luciferases, including Firefly luciferase (Fluc), *Renilla* luciferase (Rluc), and *Gaussia* luciferase (Gluc) [4,5].

GFP has certain limitations such as pH sensitivity, chloride sensitivity, and poor photostability. GFP variants improve these defective properties by site-directed mutations, increasing quantum yield, brightness, molar extinction coefficient, and photostability [6]. A series of GFP variants has been developed to expand the color palette to include blue, cyan, and yellow emission [7,8].

In these studies, enhanced GFP (EGFP) improves brightness and stability, emits better fluorescence than wild-type GFP, and can be used to monitor metabolites and quantification in cells [9]. The F64L variant of GFP (GFP2) enhances the temperature sensitivity of fluorescent proteins. Blue fluorescent protein (BFP), constructed from a targeted mutation of histidine (H) at position Y66, displays an excitation peak at 384 nm while emitting at 448 nm and can be used for cellular imaging and fluorescence resonance energy transfer (FRET) [10].

Fluc is an enzyme that catalyzes the oxidation of luciferin by oxygen from air in the presence of ATP and Mg^2+^ [11]. The reaction is accompanied by light emission in the visible region of the spectrum (540–600 nm). Firefly luciferin is a very stable and nontoxic substance [12]. This results in a very low background signal for the bioluminescence reaction, which has led to the successful application of Fluc in vitro and in vivo systems [12,13].

Although Fluc has proven to be very useful for many applications, improvements are still needed. Increasing the thermal stability of Fluc can enhance bioluminescence in vivo by increasing the effective half-life of the enzyme [14]. Protein engineering efforts have led to a number of luciferase variants with altered or improved properties such as luminescence spectral shifts, thermal stability, pH tolerance, and catalytic activity [15,16].

Rluc oxidizes coelenterazine and produces a dioxetane intermediate. Subsequently, the dioxane loses CO_2_ and produces coelenterazine in its excited state. This excited intermediate then allows the molecule to return to its ground state by emitting dissipative photons (480 nm); this enzymatic reaction does not require cofactors such as ATP [17].

The disadvantages of using natural Rluc are its rapid deactivation, blue bioluminescence, and short photoemission half-life, which limits its biotechnological applications, especially for in vivo imaging [18].

Woo et al. obtained super Rluc by replacing three amino acids (K189V, V267I, and M185V) in the Rluc sequence. super Rluc has a higher turnover rate, increased temporal output, and increased photon emission half-life when expressed in *Arabidopsis thaliana* compared to natural Rluc [19]. Loaning et al. generated Rluc with eight mutations in its structure (A55T, C124A, S130A, K136R, A143M, M185V, M253L, and S287L). This enzyme, named Rluc 8, showed a fourfold enhancement in light output and improved thermal stability compared to the natural enzyme. In addition, Rluc 8 has flash-type luminescence kinetics [18].

It is worth noting that the sensitivity of fluorescent proteins is typically lower than that of bioluminescent luciferases. However, existing bioluminescent luciferases have specific limitations. For instance, Fluc relies on cofactors [20,21], Rluc exhibits low photon production [22], and both cannot be secreted for expression. In contrast, Gluc possesses distinct characteristics, such as high sensitivity [23], natural secretion [23], compact gene fragments, non-cytotoxicity [23], short half-life [24], stable fluorescence properties [25], easy detection, and independence from cofactors like ATP and Mg^2+^ during luminescence. Consequently, Gluc finds extensive applications in monitoring promoter activity, studying protein-protein interactions, protein localization, high-throughput drug screening, and real-time monitoring of tumor occurrence and development [26,27].

Gluc, which also catalyzes bright blue light by oxidizing coelenterazine, has a bioluminescence intensity 200-fold higher than that of Fluc and Rluc, the two most widely used luciferase enzymes, and thus it is considered a potentially ideal reporter protein [23]. Attempts to improve or re-engineer the bioluminescent properties of GLuc have included extending its half-life luminescence [28,29,30] and improving the redshift of its peak luminescence at 480 nm [31,32] (the redshift is readily absorbed by tissues during in vivo application [33]).

Bioluminescence can be categorized into two types: glow-type and flash-type lu-minescence. Glow-type luminescence is characterized by its persistent and stable nature, whereas flash-type luminescence is short-lived and less stable. However, Gluc, a reporter gene, has certain limitations. One limitation is the rapid attenuation of the blue emission peak [31,34] by hemoglobin. Additionally, Gluc exhibits a flash-type bioluminescence reaction, rendering it unsuitable for high-throughput applications.

In a study conducted by John P. Welsh et al., it was observed that the M69L muta-tion in Gluc led to a decrease in specific activity [29]. The researchers also investigated the combined effects of M43L, M69L, and M110L mutations, which resulted in mutants exhibiting longer luminescence half-lives (approximately 14 min) and nearly restored enzyme specific activity to that of the wild-type Gluc [29]. Nevertheless, the precise mechanisms underlying the dynamic impact of these mutations on luminescent properties remain largely unexplored.

Mutations improve the luminescence properties of Gluc; however, the underlying kinetic mechanisms behind the experimental phenomenon that exist for mutants to affect luminescence properties have rarely been reported. Molecular dynamics simulation trajectories have been applied in recent years to the study of fluorophore enzymes such as Rluc [35], Lampyridae family’s luciferase [36]. Elucidating the kinetic mechanisms brought about by the mutations, including the conformational changes of the catalytic site and the degree of closure of the catalytic pocket, can help to reveal the effects produced by the mutant sites, determine the direction of the modification of fluorophore enzymes, and provide clues for the further design of stable and efficient fluorophore enzymes.

In this study, molecular dynamics (MD) simulations of 450 ns were combined with Markov models and neural relationship inference to explore the dynamic changes in bioluminescent properties affected by mutations in the glow-type mutant GlucM1 and the flash-type mutant GlucM2, using wild-type Gluc (GlucWT) as a control. Divide the conformational changes of different mutants combined with substrates in six systems into two groups for analysis, the first comprising the blank proteins with no substrate bound (Free-GlucWT, Free-GlucM1, Free-GlucM2), and the second comprising the complexes following substrate binding (GlucWT-CTZ, GlucM1-CTZ, GlucM2-CTZ). Our research provides a theoretical basis for exploring the mechanism by which Gluc mutations affect bioluminescence properties and provides some useful clues for designing Gluc.

## 2. Results

### 2.1. Protein Preparation and Structure Stability

Gluc is an exceptional enzyme comprising nine α-helices [37]. Its structure is characterized by two repetitive sequences that form bundles of four helices linked by three disulfide bonds. The initial helix exhibits tight interactions with the other helices. Notably, Gluc possesses a unique cavity located between α1, α4, and α7, composed of 19 specific residues: N10, V12, A13, V14, S16, N17, F18, L60, S61, I63, K64, C65, R76, C77, H78, T79, F113, I114, and V117. This cavity serves as the binding site for CTZ, playing a vital role in Gluc’s bioluminescent activity [37].

**Figure 1:**
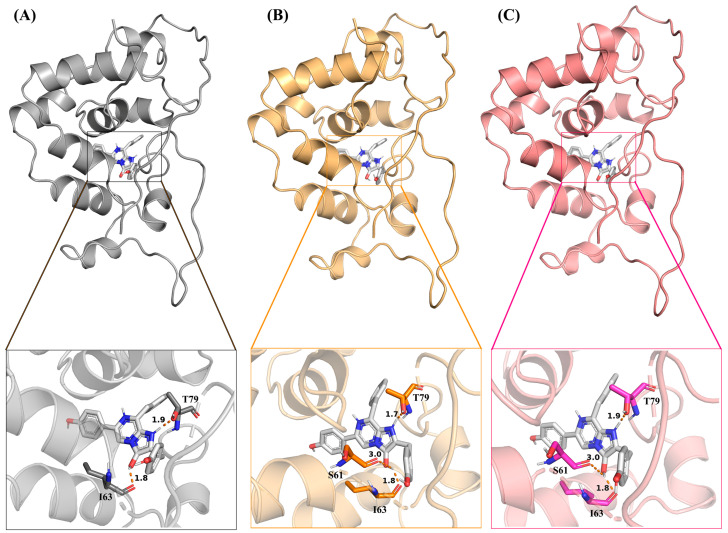
(**A**) The hydrogen bonds (T79, I63) between GlucWT and CTZ. (**B**) The hydrogen bonds (T79, I63, S61) between GlucM1 and CTZ. (**C**) The hydrogen bonds (T79, I63, S61) between GlucM2 and CTZ.

Figure 1A–C displays the hydrogen bonding patterns between GlucWT, glow-type mutant GlucM1, flash-type mutant GlucM2, and the substrate CTZ. Molecular docking was employed to examine the binding of CTZ to these variants. GlucWT forms two hydrogen bonds (T79, I63) with CTZ, while GlucM1 and GlucM2 exhibit three hydrogen bonds (T79, I63, S61) each with CTZ. Notably, there is an increased number of hydrogen bonding interactions between the glow-type and flash-type mutants and CTZ. Additionally, the glow-type mutant GlucM1 demonstrates a shorter bond length and stronger interaction. The stability of the complexes is positively correlated with the number of hydrogen bonds.

**Figure 2:**
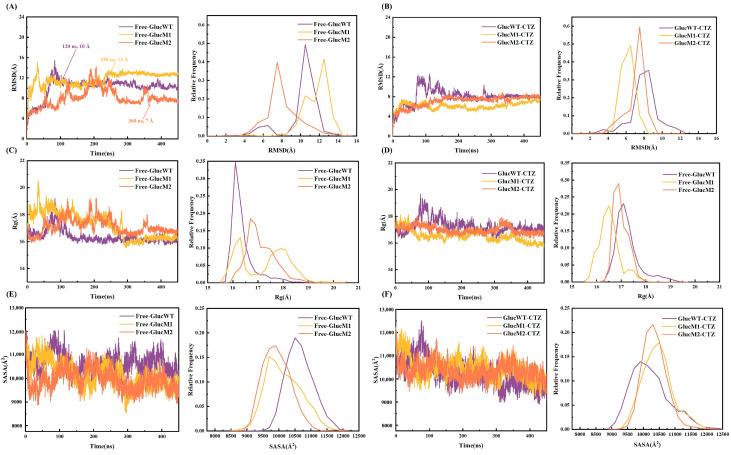
(**A**) The RMSD diagrams of Free-GlucWT, Free-GlucM1, and Free-GlucM2 (the arrows mark the approximate stabilization nodes). (**B**) The RMSD diagrams of GlucWT-CTZ, GlucM1-CTZ, and GlucM2-CTZ. (**C**) The R_g_ diagrams of Free-GlucWT, Free-GlucM1, and Free-GlucM2. (**D**) The R_g_ diagrams of GlucWT-CTZ, GlucM1-CTZ, and GlucM2-CTZ. (**E**) The SASA diagrams of Free-GlucWT, Free-GlucM1, and Free-GlucM2. (**F**) The SASA diagrams of GlucWT-CTZ, GlucM1-CTZ, and GlucM2-CTZ.

To assess the convergence of each system and ensure the subsequent sampling strategies’ reliability, the root mean square deviation (RMSD) of Cα atoms was calculated (Figure 2A,B). The Free-GlucWT system achieves equilibrium in approximately 120 ns, exhibiting fluctuations at around 10 Å. Similarly, Free-GlucM1 reaches equilibrium after approximately 250 ns, stabilizing at around 13 Å. Free-GlucM2, on the other hand, attains equilibrium after approximately 360 ns, with a stable RMSD of approximately 7 Å. In contrast, the remaining three systems, GlucWT-CTZ, GlucM1-CTZ, and GlucM2-CTZ, reach equilibrium at approximately 300 ns and stabilize at approximately 8 Å. Notably, the RMSD analysis indicates that the glow-type mutant GlucM1 in the free protein achieves stability at the fastest rate, and all systems reach stability more rapidly after binding to the substrate CTZ. We have also performed the calculation of the RMSD of the ligand CTZ in the complex, and it was done with the binding sites aligned, and the results showed that the ligand CTZ of GlucWT, GlucM1, and GlucM2 fluctuated within 2 Å and eventually converged stably around 4 Å (Figure S5).

The radius of gyration (Rg) was calculated to assess protein compactness changes in the six systems during the simulation process. Comparing Free-GlucWT, it is observed that the Rg values of Free-GlucM1 and Free-GlucM2 increase. However, the Rg spectrum of Free-GlucM1 is broader, suggesting a conformational change following the mutation (Figure 2C,D). Upon binding with the substrate CTZ, GlucM1-CTZ and GlucM2-CTZ exhibit decreased Rg values, and their Rg spectra are narrower than that of GlucWT-CTZ, indicating a more stable conformation in the complex system after the mutation.

Furthermore, the solvent-accessible surface area (SASA) value of the free protein fluctuates around 1000 Å^2^ after 350 ns. Compared to GlucWT-CTZ, the SASA value of GlucM1-CTZ increases and stabilizes at approximately 10,750 Å^2^, indicating enhanced protein hydrophilicity due to the glow-type mutation. In contrast, the SASA spectrum of GlucM2-CTZ is narrower, suggesting that the flash-type mutations lead to a more hydrophilic conformation (Figure 2E,F).

In summary, after a 450 ns MD simulation, all six systems exhibit stability and can be utilized for further research.

### 2.2. Dynamical Cross-Correlation Matrix Analysis

**Figure 3:**
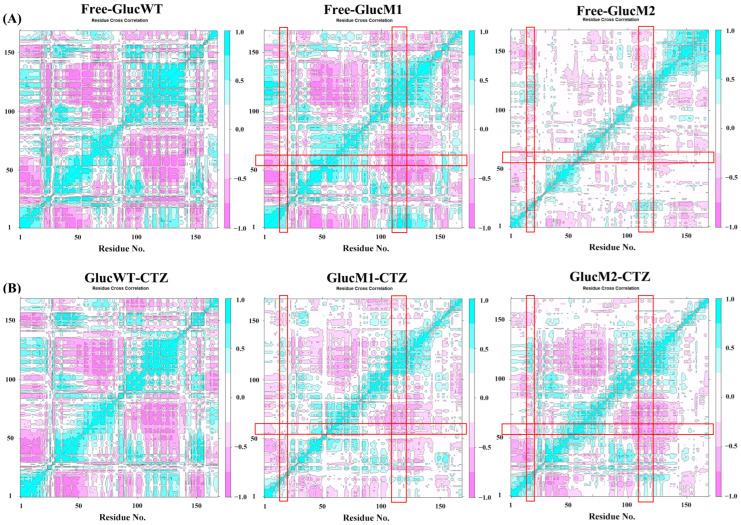
(**A**) The Dynamical Cross-Correlation Matrix diagrams of Free-GlucWT, Free-GlucM1, and Free-GlucM2. (**B**) The Dynamical Cross-Correlation Matrix diagrams of GlucWT-CTZ, GlucM1-CTZ, and GlucM2-CTZ. The intersection of the red rectangles shows the motions between α1, α4, and α7 in the active site.

Figure 3 depicts the dynamic cross-correlation matrix (DCCM) analysis for all Cα atoms. Positive regions are shown in cyan and negative regions in pink, indicating correlated and anti-correlated motions between residue Cα atoms. Low correlations between residue motions indicate uncorrelated motions. The intersection of the red rectangles shows the motions between α1, α4, and α7 in the active site. As can be observed from Figure 3, the pink areas indicate inverse motions between α1, α4, and α7 in all systems. This is consistent with previous results that the distance between α1, α4, and α7 in Gluc decreases.

### 2.3. Molecular Mechanics/Poisson–Boltzmann Surface Area Calculation

The Molecular Mechanics/Poisson–Boltzmann Surface Area (MM/PBSA) approach has been widely applied as an efficient and reliable free energy simulation method to model molecular recognition, such as for protein-ligand binding interactions. To calculate the free energy between GlucWT, GlucM1, GlucM2, and CTZ, we utilized the MMPBSA.py program within the AmberTools22 software package [38]. The obtained results are presented in Table 1, Table 2 and Table 3.

In Table 1, Table 2 and Table 3, “Average” represents the energy mean, “Std. Dev.” represents the standard deviation, and “Std. Err. of Mean” represents the standard error of the sample mean. In the energy components section, ∆E_ele_ is the electrostatic energy; ∆E_vdw_ is the van der Waals energy; ∆G_gas_ is the molecular mechanics term (energy in the gas phase), which is equal to ∆E_ele_ plus ∆E_vdw_; ∆G_sol_ is the solvation energy, which incorporates the change in internal energy and entropy of the solute into the solvent, as well as the work required to displace the solvent; and ∆G_total_ is equal to ∆G_sol_ plus ∆G_gas_.

Table 1, Table 2 and Table 3 show that GlucWT and GlucM1 are comparable in terms of van der Waals energies, both of which are larger than GlucM2, while GlucM2 has larger electrostatic force than GlucM1 and GlucWT is the smallest. According to Table 1, Table 2 and Table 3, MM/PBSA analysis reveals that GlucM1-CTZ exhibits the lowest binding free energy and the highest affinity, followed by GlucWT and GlucM2. 

**Figure 4:**
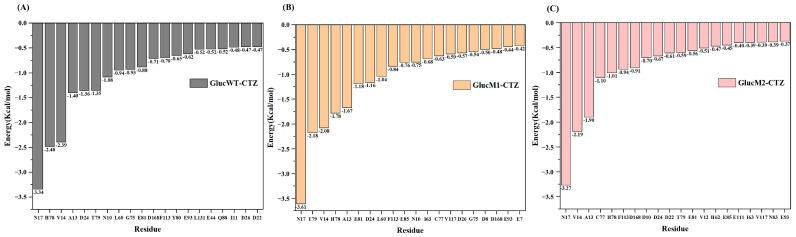
(**A**) The residue contribution map of GlucWT-CTZ. (**B**) The residue contribution map of GlucM1-CTZ. (**C**) The residue contribution map of GlucM2-CTZ.

Figure 4A–C shows the binding energy contributions of residues in the three complex systems. As can be seen in Figure 4A, N17, H78, V14, A13, D24, and T79, among others, are key residues for the binding of GlucWT to CTZ. N17, T79, V14, H78, and A13, among others, are key residues for the binding of GlucM1 to CTZ (Figure 4B). In contrast, N17, V14, A13, C77, and H78 were key residues for GlucM2 binding to CTZ (Figure 4C). In conclusion, the contribution results of residues in Figure 4 highlight that the binding of Gluc and CTZ is primarily influenced by the residue N17, which plays a significant role, in addition to the important contributions of V14, A13, and H78.

### 2.4. Conformational Changes during MD Simulations

**Figure 5:**
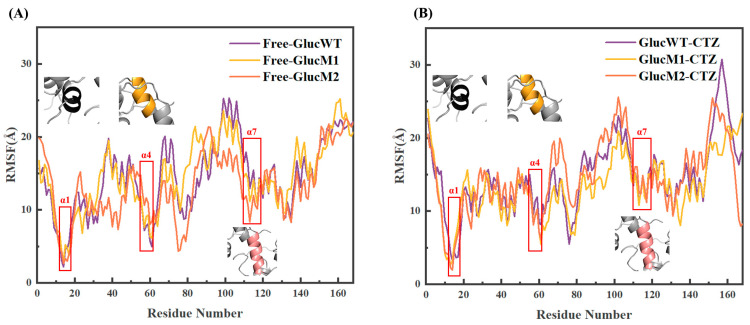
(**A**) The RMSF results of Free-GlucWT, Free-GlucM1, and Free-GlucM2. (**B**) The RMSF results of GlucWT-CTZ, GlucM1-CTZ, and GlucM2-CTZ (the protein conformation diagram in the figure represents the structural situation at this site).

To assess the impact of mutations on Gluc, we calculated the root mean square fluctuation (RMSF) of Cα atoms (Figure 5A,B). Generally, the six systems exhibit similar RMSF distributions, with a few exceptions. Our focus lies on observing RMSF changes at α1, α4, and α7, which constitute the active sites. Overall, the free protein system displays a certain level of structural fluctuation, with Free-GlucWT exhibiting greater flexibility compared to Free-GlucM1 and Free-GlucM2. Upon binding with the substrate CTZ, all systems demonstrate comparable flexibility at α1, α4, and α7, indicating relatively stable structural fluctuations at the active site. These results suggest that the binding of the substrate CTZ to luciferase Gluc, including its mutants, may enhance their interaction with α1, α4, and α7.

**Figure 6:**
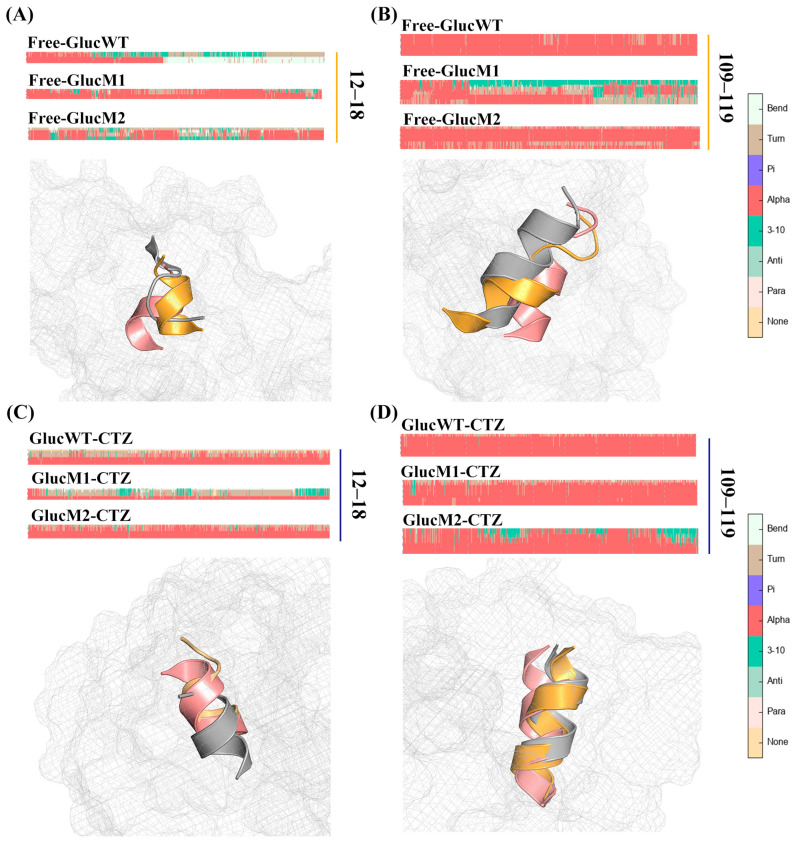
(**A**) The secondary structure changes’ probability of Free-GlucWT, Free-GlucM1, and Free-GlucM2 in residues 12–18. (**B**) The secondary structure changes’ probability of Free-GlucWT, Free-GlucM1, and Free-GlucM2 in residues 109–119. (**C**) The secondary structure changes’ probability of GlucWT-CTZ, GlucM1-CTZ, and GlucM2-CTZ in residues 12–18. (**D**) The secondary structure changes’ probability of GlucWT-CTZ, GlucM1-CTZ, and GlucM2-CTZ in residues 109–119 (gray for GlucWT, orange for GlucM1, and pink for GlucM2).

The analysis of protein secondary structure is a crucial component of molecular dynamics simulations. Figure 6A illustrates the changes in the secondary structure of the α1 helix (residues 12–18) among the Free-GlucWT, Free-GlucM1, and Free-GlucM2 systems. Additionally, Figure 6B depicts the 3D structural variations of the α7 helix (residues 109–119) in these conformations (represented by gray for Free-GlucWT, orange for Free-GlucM1, and pink for Free-GlucM2). Similarly, Figure 6C showcases the variations in the secondary structure of the α1 helix (residues 12–18), and Figure 6D displays the 3D structure of the α7 helix (residues 109–119) among the GlucWT-CTZ, GlucM1-CTZ, and GlucM2-CTZ systems (represented by gray for GlucWT-CTZ, orange for GlucM1-CTZ, and pink for GlucM2-CTZ).

Observing Figure 6A, Free-GlucM1 exhibits the highest degree of α-helical structure within the residue domain of 12–18, followed by Free-GlucM2, while Free-GlucWT displays the lowest degree of helicity. Similarly, in the presence of CTZ, GlucM2-CTZ demonstrates the most α-helices, followed by GlucWT-CTZ, and finally GlucM1-CTZ (Figure 6C). Furthermore, Figure 6C highlights that Free-GlucWT has the highest degree of α-helical structure within the residue domain of 109–119, followed by Free-GlucM2, while Free-GlucM1 exhibits the lowest degree of helicity. In contrast, the α-helical structure of GlucM1 significantly increases upon binding CTZ. Notably, the α4 helices in all systems remain stable.

The Markov model calculation process was conducted using the pyemma software package (version number: 2.5.12) [39]. For instance, in the case of Free-GlucWT, k-means clustering of the trajectories was initially performed, and the k-value yielding the highest score was selected based on the variational approach for Markov processes (VAMP-2) scoring results (Figure S1). To determine the appropriate lag time, Figure S2 was examined, revealing the convergence of the relaxation time scale for the entire system at a lag time of 1.2 ns. Consequently, a lag time of 1.2 ns (equivalent to 12 steps considering the 0.1 ns step size used for molecular simulation trajectories) was chosen for the system. The results of the ck test on the Markov model, presented in Figure S3, indicate favorable Markov properties of the constructed model, establishing its applicability for subsequent research.

**Figure 7:**
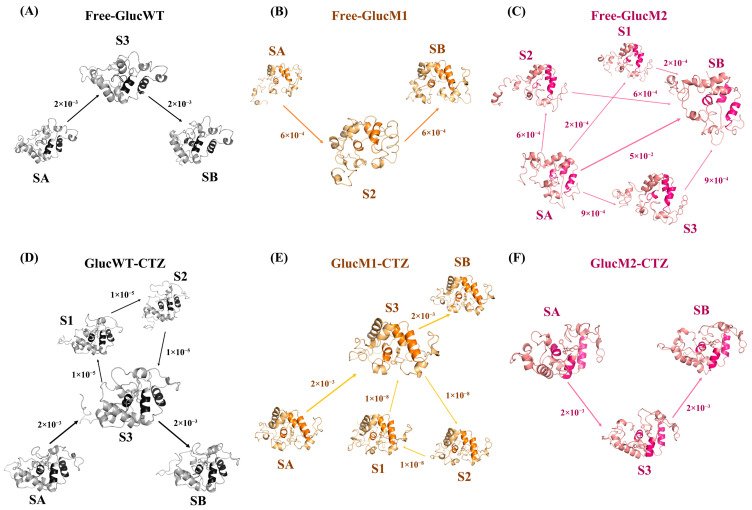
Flux analysis of Free-GlucWT (**A**), Free-GlucM1 (**B**), Free-GlucM2 (**C**), GlucWT-CTZ (**D**), GlucM1-CTZ (**E**), and GlucM2-CTZ (**F**).

The transition probability matrix of the Markov model was utilized for flux analysis, and the outcomes are depicted in Figure 7A, with the highlighted labeled region focusing on the α-helix area under investigation. By analyzing the flux and the proportional representation of each pathway, Table S1 was generated. SA represents the initial state of the Markov model, SB represents the final state of the Markov model, and S1, S2, and S3 all represent the transition state in the process of transition from the initial state to the final state. The labels 1, 2, and 3 are used to distinguish different microstates, and there is no order of precedence. These microstates are defined and labelled after Perron Cluster Cluster Analysis (PCCA) clustering by the Markov model, and microstates with the same name are different in different systems.

Upon examining the flux result, it becomes evident that the predominant conformational transition pathway is Free-GlucWT: SA→S3→SB, accounting for 100% of the total flux (Figure 7). Thus, considering the relationship between secondary structure changes and the flux analysis findings, it can be observed that the most representative Free-GlucWT: SA→S3→SB conformational transition primarily involves the formation of the α1 helix (Figure S4).

Cluster analysis, Markov model construction, and flux analysis were similarly carried out for Free-GlucM1, Free-GlucM2, GlucWT-CTZ, GlucM1-CTZ, and GlucM2-CTZ. The results are presented in Figure 7B–F and Table S1. In the case of Free-GlucM1, the most representative conformational transition pathway is Free-GlucM1: SA→S2→SB, accounting for 100% of the total flux (Figure 7). Notably, Free-GlucM1 primarily involves the formation of the α4 and α7 helical regions (Figure S4). Conversely, for Free-GlucM2, the predominant Free-GlucM2: SA→SB conformational transition mainly encompasses the partial formation of the α1 helix (Figure S4). Regarding GlucWT-CTZ, no substantial changes in helix extent are observed in the most representative GlucWT-CTZ: SA→S3→SB conformational transition (Figure S4).

Examining GlucM1-CTZ, the pathway with the highest probability, GlucM1-CTZ: SA→S3→SB, reveals that upon CTZ binding, the α1 helix undergoes partial formation and subsequent de-helixing (coinciding with the extent of the α1 helix in GlucM1’s secondary structure). In the case of GlucM2-CTZ, the pathway with the highest probability, GlucM2-CTZ: SA→S3→SB, demonstrates partial de-helixing of the α7 helix upon CTZ binding (matching the extent of the α7 helix in GlucM1’s secondary structure) and a contraction of the active pocket (Figure S4). The partial formation of the α7 helix compared to GlucM1 and GlucM2, along with conformational changes in both the α1 and α4 helices, suggests a plausible connection between the α-helix conformational changes and the relevant luminescent properties.

### 2.5. Distance Analysis

**Figure 8:**
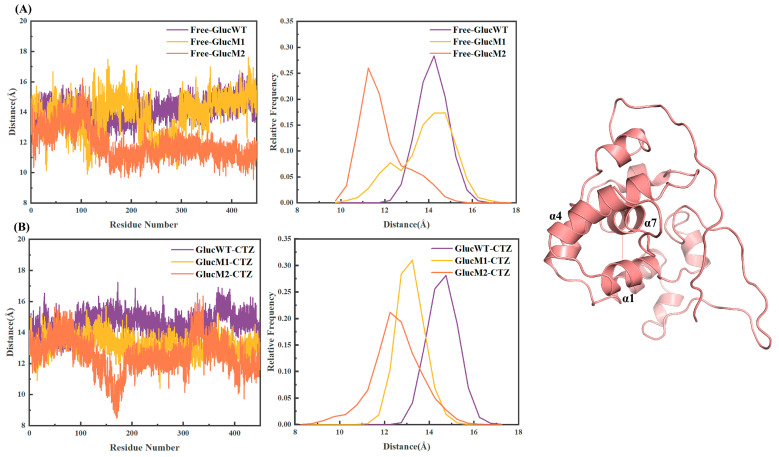
(**A**) The distance of Free-GlucWT, Free-GlucM1, and Free-GlucM2. (**B**) The distance of GlucWT-CTZ, GlucM1-CTZ, and GlucM2-CTZ.

To investigate the conformational changes in the active site during CTZ binding, the distance between α1 and α7 (with α4 exhibiting greater stability) was measured and analyzed through a 450 ns molecular dynamics simulation. Figure 8 illustrates that upon CTZ binding, the GlucM1 active pocket undergoes significant closure, shrinking from approximately 14.8 Å to around 12.8 Å. In comparison, GlucM2 displays a relatively more open state, transitioning from 11.5 Å to 12.5 Å, as opposed to Free-GlucM2. Notably, the Free-GlucM2 pocket exhibits a more closed configuration compared to Free-GlucWT. Conversely, the GlucWT activity pocket does not exhibit significant changes, maintaining a size of approximately 14.3 Å to 14.8 Å following CTZ binding.

**Figure 9:**
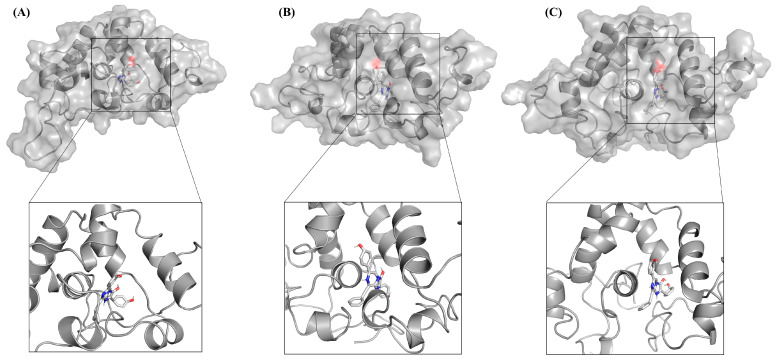
The representative conformational diagrams of GlucWT-CTZ (**A**), GlucM1-CTZ (**B**), and GlucM2-CTZ (**C**).

By comparing the conformational diagrams of the structures with the highest clustering, it was observed that upon CTZ binding, the active sites exhibited varying degrees of closure: GlucM1 > GlucM2 > GlucWT (Figure 9). This suggests that the mutation in the glow-type mutant GlucM1 is more favorable for substrate binding, while the mutation in the flash-type mutant GlucM2 is comparatively less stable.

### 2.6. Neural Relationship Inference

**Figure 10:**
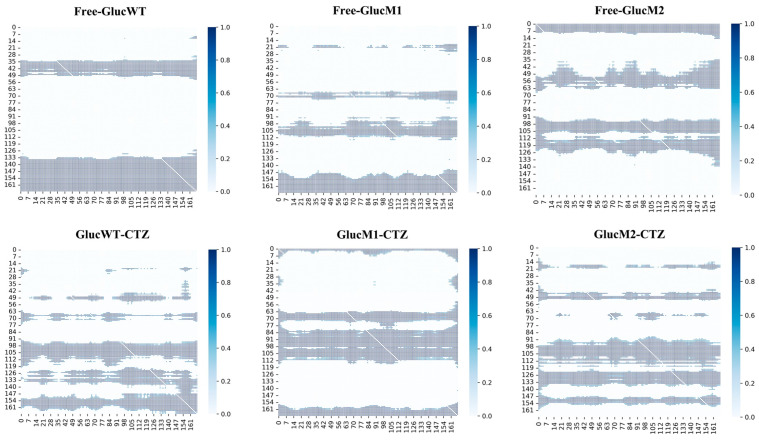
**The** NRI results of Free-GlucWT, Free-GlucM1, Free-GlucM2, GlucWT-CTZ, GlucM1-CTZ, and GlucM2-CTZ.

The NRI model was trained using the encoder and decoder on MD trajectories, enabling us to analyze the distribution of learned edges between residues (Figure 10). Notably, frequent occurrences of learned edges were observed between residues 96–110 and other residues, indicating the significance of residues 96–110 in protein movement. Upon CTZ binding, GlucM1 exhibited a more concentrated presence of key residues for protein movement within the middle residues 84–112, while the edges associated with residues 147–154 vanished. In contrast, GlucM2 displayed a higher frequency of learned edges between residues 91–112 and newly observed edges at residues 147–161. These changes in the learned edges highlight the alterations and distinctions in key structural elements influencing protein motility following substrate CTZ binding.

## 3. Discussion

Luciferase (Luc) is an enzymatic protein responsible for catalyzing substrate oxidation, commonly known as luciferin. It is frequently utilized as a reporter protein alongside GFP [15,40,41]. *Gaussia* luciferase (Gluc) is a luciferase variant obtained from the marine organism *Gaussia princeps* [42]. Gluc generates vibrant blue light through the oxidation of CTZ. Despite its small size, with a molecular weight of 18.2 kDa (excluding secretion labels), Gluc exhibits remarkably strong bioluminescence intensity, surpassing the two most widely used luciferases, Fluc and Rluc, by 200 times. This characteristic positions Gluc as a promising candidate for an ideal reporter protein in various fields, including molecular biology, oncology, immunology, microbiology, and more.

Nan Wu et al. conducted a study utilizing heteronuclear nuclear magnetic resonance to elucidate the solution structure of Gluc and infer the binding cavity for the fluorescent substrate CTZ [37]. Their findings revealed that Gluc is composed of nine alpha helices, forming two antiparallel bundles through homologous sequences with consecutive repeats. These bundles are interconnected by three disulfide bonds, resulting in a distinct cavity situated between the central helices α1, α4, and α7. Within this cavity, 19 residues, including N10, V12, A13, and V14, play a vital role in constituting the CTZ binding site, which is crucial for Gluc’s bioluminescent activity.

Efforts to enhance or redesign the bioluminescence properties of Gluc have involved various approaches. These include extending its luminescence half-life [27,29,43] and improving the luminescence peak redshift to 480 nm [32,44], which is more effectively absorbed by tissues during in vivo applications [33]. Maguire et al. [45] presented a mutant Gluc with a prolonged luminescent signal. This mutant carried a single-point mutation (M43I) that extended the luminescence intensity half-life from 2.4 to 9.1 min when tested in vitro using purified enzyme in the presence of the detergent Triton X-100. However, it’s worth noting that this mutant exhibited a reduction in specific activity of approximately threefold.

In a study conducted by John P. Welsh et al., it was observed that the mutation M69L in the Gluc sequence led to a significant decrease in specific activity. Conversely, the same mutation at position 110 retained most of the enzyme’s specific activity but exhibited similar explosive dynamics to the wild-type enzymes [29]. Conversely, the M43L mutation resulted in a notable decrease in specific activity but extended the light emission with a half-life of over 8 min, which was more than six times longer than the natural Gluc signal (*p* < 0.005). This mutation appeared to stabilize the dynamic signal of Gluc. The researchers also examined the combined effects of M43L, M69L, and M110L mutations in Gluc. The results demonstrated that the combination of these mutations resulted in mutants with prolonged luminescence half-lives (*p* < 0.005), lasting approximately 14 min. Additionally, the mutant nearly restored the same enzyme-specific activity as the wild-type enzyme [29].

The significance of the conformational flexibility of a loop-helix fragment of Rluc for ligand binding was demonstrated by Andrea Schenkmayerova et al. The transplantation of this dynamic fragment has been shown to reduce product inhibition and produce highly stable glow-type bioluminescence [46].

To investigate the relationship between the structural changes induced in AncHLD-RLuc (that was a particularly stable ancestral protein reconstructed from the catalytically distinct but evolutionarily and structurally related haloalkane dehalogenases (HLD) and Rluc) by Insertion-deletion (InDel) mutagenesis and enhanced kinetics of substrate binding, Andrea Schenkmayerova et al. crystallised AncINS and compared its structure to previously published structures of AncHLD-RLuc and RLuc8. These proteins differ in their cap domains. Important changes were identified in the conformation of the α4 helix and L9 loop in the cap domain, the size of the active site cavity, the width of the tunnel mouth, and the active site accessibility. The asymmetric unit of the crystal lattice of AncINS contains two monomers (chains A and B). Chain A displays a structural similarity to the AncHLD-RLuc template but features a π-helix bulge in the α4 helix, where the L162 insertion and F163P substitution occurred. In contrast, the α4 helix in AncINS chain B exhibits a marked distortion towards the α5 helix. RLuc8 also contains two monomers within the asymmetric unit. Chain B is similar to that of AncHLD-RLuc, whereas chain A is in an open conformation with the α4 helix oriented away from the α5 helix [46].

The results of this study showed that, from the perspective of the whole system, the stability of GlucM1-CTZ was higher than that of GlucWT-CTZ and GlucM2-CTZ, which may be due to more hydrogen bonds and shorter bond lengths between the glow-type mutant M1 and CTZ (as shown in Figure 1), which is in line with the reported higher specific activity of M1 (M43L, M69L, M110L) [29]. Similarly, GlucM2-CTZ demonstrates greater stability than GlucWT-CTZ, with an increased number of hydrogen bonds between the GlucM2 mutant and the substrate, as illustrated in Figure 1. The key residues T79, I63, and S61 shown in the docking results are all residues that make up the active site, and it has been shown that S61 is 92% conserved among them [37]. 

Analyzing the entire molecular dynamics simulation process, it can be inferred that the binding cavity formed by α1, α4, and α7 in GlucM1 achieves the highest degree of closure upon substrate CTZ binding, followed by GlucM2 and finally GlucWT, as illustrated in Figure 8. These differences in the degree of active site closure after substrate binding contribute to variations in system stability, consistent with the results in Figure 2 that the RMSD and Rg values of GlucM1 were lowest after binding CTZ, while the system stability affected the luminescent properties such as the specific activity and half-life of the enzyme. The substitution of methionine with leucine resulted in a higher hydrophilic index of the amino acid, leading to a more hydrophilic complex upon substrate binding by the mutant [47], consistent with the SASA results in Figure 2.

Figure 3 shows that the pink area indicates reverse motion between α1, α4, and α7 in all systems. This means that the distance between α1, α4, and α7 in Gluc decreases during the simulation, and the pocket shrinks.

Table 1, Table 2 and Table 3 show that GlucM1-CTZ has the lowest binding free energy, which is in line with the reported higher specific activity of M1 (M43L, M69L, M110L) [37]. Furthermore, during the binding process, the residue N17, which contributes significantly, was found to possess a weakly conserved type [37]. Other residues providing significant contributions in the MM/PBSA results (V14, A13, H78) were all confirmed to be residues comprising the active site, consistent with experimental results [37]. 

Upon binding CTZ, the active pocket of the glow-type mutant GlucM1 exhibited significant closure, resulting in a smaller distance between α1 and α7, as depicted in Figure 7 and Figure 8. Conversely, the active pocket of the flash-type mutant GlucM2 appeared more open compared to the unbound protein, leading to an increased distance between α1 and α7, as shown in Figure 7 and Figure 8. This observation may be attributed to the mutation causing a weaker substrate-binding interaction force and a less tightly bound substrate. It is worth noting that the closure state of both mutants is superior to that of GlucWT. Distance analysis and conformational analysis at the active pocket are also consistent with the DCCM results, as can be observed in Figure 3A, where the pink area shows that both combinations of α4, α1 and α4, α7 are kinematically anticorrelated in Free-GlucM1, whereas in Figure 3B, the pink color becomes lighter, suggesting that the degree of anticorrelation decreases upon binding of the substrate CTZ. For GlucM2, the degree of motor anticorrelation was elevated upon binding the substrate CTZ. The conformational change at the active pocket after the mutation corroborates with the notion that the catalytic site of Gluc consists mainly of a cavity formed by α1,4,7 in the study of Nan Wu et al. [38] These findings suggest that mutations with distinct bioluminescent properties induce conformational changes at the active site, influencing the interaction between Gluc and the substrate. 

Gluc and Rluc are the same marine biological luciferase with in vivo luminescence advantage and the same luminescent substrate (CTZ) [17,48]. It has been confirmed that mutations lead to disruption of secondary structure and perturbation of folding pathways [49]. Through main chain mutation and other steps, Andrea Schenkmayerova et al. [37] established an optimal variant of the α4 helix with a 124-fold increase in catalytic efficiency relative to the ancestral enzyme and a molecular probe with a glow-type bioluminescence. Moreover, this variant is conformationally flexible relative to the ancestral enzyme, focusing mainly on structural elements at key sites (L9 and L14 loops and the α4 helix), which increases the rate of the initial collision step, resulting in faster kinetics of substrate binding.

As CTZ is a fluorescent substrate, affecting the binding of CTZ at the active site will affect the luminescence of fluorescein enzyme, and protein structure determines function, it is reasonable to deduce that the GlucM2 mutant has an open conformation and does not bind tightly, as well as changes in stability before and after the binding itself, resulting in a short half-life and flash. The GlucM1 mutant has a closed conformation and binds tightly, resulting in a high activity with a long half-life, and presents a glow. The GlucM1 mutant has a closed conformation and is tightly bound, resulting in a long half-life and a glow.

The results obtained from Markov modeling (Figure 7, Table S1) revealed that the degree of conformational helicity in the GlucWT-CTZ system, used as a control, remained relatively unchanged. Analyzing the most representative conformational transition pathway of GlucM1-CTZ (SA→S3→SB), it was observed that the glow-type mutation had a high probability of leading to the formation of the S3 (GlucM1-CTZ) microstate in GlucM1. This conformational change involved partial helicalization of the α1 helix, followed by de-helicalization upon binding of CTZ. This finding aligns with the decrease in helix formation observed in the secondary structure analysis of GlucM1-CTZ compared to Free-GlucM1 (Figure 6). 

Likewise, the primary conformational transition pathway of GlucM2-CTZ (SA→S3→SB) suggested that the flash-type mutation had a notable likelihood of inducing the formation of the S3 (GlucM2-CTZ) microstate in GlucM2. Upon CTZ binding, there was partial de-helicalization observed in the α7 helix, accompanied by a constricted state in the active pocket, which aligned with the decreased degree of helicality observed in GlucM2-CTZ during the analysis of secondary structure (Figure 6). Furthermore, the extent of active pocket closure (GlucM1 > GlucM2 > GlucWT) upon CTZ binding was linked to the conformational alterations in helix formation within the glow-type and flash-type mutants (Figure 8 and Figure 9). The findings derived from the flux analyses of the Markov model yielded valuable insights into the connection between bioluminescence characteristics and conformational changes within the mutants. The conformational change at the active pocket after the mutation corroborates with the notion that the catalytic site of Gluc consists mainly of a cavity formed by α1,4,7 in the study of Nan Wu et al. [38] Different mutation sites may lead to conformational changes in different parts, which in turn affect the luminescent properties of Gluc.

NRI indicates that after binding to CTZ, the key residues for protein movement in GlucM1 were more centred on central residues 84–112. And GlucM2 had an increased frequency of learnt edge at residues 91–112 (Figure 10). The fact that Q112 is involved in stabilising hydrogen bonds has been shown [17]. In addition, previous mutational analyses have shown that F151 also plays an important role in GLuc activity [37].

Molecular dynamics simulations have the capability to track the position and movement of individual atoms at each time step [50]. Markov models, on the other hand, enable analysis of conformational change pathways during the binding processes of small molecules [51]. Neural relational inference (NRI) models can be utilized to infer potential interactions and explore protein denaturation processes as dynamic networks of interacting residues [52]. By combining molecular dynamics simulations, Markov models, and NRI analysis, researchers can gain insights into the relationship between bioluminescence characteristics and conformational changes in Gluc mutants. This integrated approach holds promise in providing valuable clues for the subsequent design and improvement of reporter genes.

## 4. Materials and Methods

### 4.1. System Preparation

To investigate the correlation between bioluminescent properties and conformational changes in Gluc, we conducted an analysis using six different systems categorized into two groups. The first group consisted of the wild-type Free-GlucWT, the glow-type mutant Free-GlucM1, and the flash-type mutant Free-GlucM2. The second group included the wild-type complex GlucWT-CTZ, the glow-type mutant complex GlucM1-CTZ, and the flash-type mutant complex GlucM2-CTZ. The 3D structure of Gluc (PDB code: 7d2o) [37] was retrieved from the Protein Data Bank (www.rcsb.org). We used Discovery Studio 2019 [53] to remove water and ligands from the protein, resulting in the formation of Free-GlucWT. Chimera 1.14 was employed to introduce mutations in Gluc proteins, generating the glow-type mutant GlucM1 (M43L, M69L, M110L) and the flash-type mutant GlucM2 (M69L).

Using the crystal structure obtained from the PDB database, we determined the active site’s position. The structures we aimed to dock were GlucWT-CTZ, GlucM1-CTZ, and GlucM2-CTZ. Autodock Tools 1.5.6 [54] includes the AutoGrid program, which calculates relevant energies at lattice sites, and the AutoDock program, which performs conformational search and evaluation with diverse applications. For molecular docking, we defined the coordinates and radius of the docking box as (4.01, 0.53, −0.73, and 14.34) and conducted the docking procedure using Autodock. The docking box involved the following residues: N10, V12, A13, V14, S16, N17, F18, L60, S61, I63, K64, C65, R76, C77, H78, T79, F113, I114, and V117. This process resulted in the generation of complex structures, namely GlucWT-CTZ, GlucM1-CTZ, and GlucM2-CTZ.

### 4.2. Molecular Dynamic Simulation

Molecular dynamics (MD) is one of the most important theoretical tools in biology, based on Newton’s second law and the calculation of equations of motion to construct and analyse the system to be investigated. MD can explore the key information of protein motion and capture the displacement changes of all particles in the system in femtoseconds, which is difficult to achieve by any experimental means nowadays. Therefore, molecular dynamics simulations (MD) are widely used to explain and expand experimental information.

In this experiment, we conducted 450 ns molecular dynamics simulations on six systems using the Amber 22 software [37]. The systems included the wild-type Free-GlucWT, glow-type mutant Free-GlucM1, flash-type mutant Free-GlucM2, as well as the wild-type complex GlucWT-CTZ, glow-type mutant complex GlucM1-CTZ, and flash-type mutant complex GlucM2-CTZ. 

The application of MD contains several aspects of protein and small molecule preparation, complex topology, and coordinate establishment, simulation task setting and submission. Proteins were prepared using the pdb4amber program; the small molecule was first generated using antechamber to generate a small molecule parameter file (mol2 file) and then parmchk2 to generate a frcmod file. The complex topology and coordinates were established using the ff99SB force field [55,56] for proteins, and the water box force field used in the simulation was the tip3p force field [57,58].

Once the systems were constructed, we performed energy minimization to eliminate atomic collisions in the initial structure. The energy-minimization process consisted of two steps: the most rapid descent method and the conjugate gradient method. The maximum number of cycles for minimization is 1000, and the initial 0 to 500 cycles use the most rapid descent algorithm and the 500 to 1000 cycles use the conjugate gradient algorithm. After energy minimization, the initial system structure was stabilized, and the simulated temperature was gradually increased from 0 K to 300 K over a period of 50 ps. The total number of simulation steps run was 25,000 with a simulation step size of 0.002 ps, SHAKE was enabled to constrain all the bonds that contain hydrogen, equivariant periodic boundaries were enabled, the temperature was controlled using a Langevin thermostat, and the collision frequency was set at 2.0. The trajectory information was written to the trajectory file once every 500 steps. After reaching the desired temperature, the system was allowed to re-equilibrate for 50 ps to achieve density equilibrium (NVT equilibrium). Subsequently, the system was equilibrated at 300 K for 500 ps at constant pressure (NPT equilibrium) under the NPT tether. The pressure was controlled using a Berendsen thermostat, the thermostat temperature was maintained at 300 K, and the coordinates and velocities were read from an unformatted inpcrd coordinate file using periodic boundary conditions at constant pressure, with the energy information written to the Amber output file (.out) once every 1000 steps and the current trajectory written to the Amber trajectory file once every 1000 steps. This constant pressure equilibration step ensured that the system reached its final equilibrium state. A total of 250,000 steps were performed with a step size of 0.002 ps. Once the thermodynamic parameters were stabilized, molecular dynamics simulations were performed on the six systems for a duration of 450 ns. A total of 4500 frames were recorded for each system and saved for further analysis and study.

### 4.3. Trajectory Analysis

The trajectory analysis encompassed several parameters, including RMSD, Rg, SASA, RMSF, and distance analysis. These calculations were performed using the cpptraj module from Amber22 [59]. Additionally, the secondary structural data was obtained using the cpptraj module and visualized through gnuplot 5.2 and Pymol 2.5.4 [60]. DCCM-PCA calculations were conducted using R-STUDIO (R 4.2.1) [61].

### 4.4. MM/PBSA Calculations

The MM/PBSA method is a compromise between accuracy and speed and is widely used in receptor-ligand binding free energy calculations. The full name of the method is Molecular Mechanics Poisson–Boltzmann Surface Area. The basic principle of MM/PBSA is to calculate the difference between the binding free energies of two solvated molecules in the bound and unbound states or to compare the free energies of the same molecule in different solvated conformations.

The binding free energy (∆G_bind_) can be expressed as follows

(1) $$\begin{array}{c} {∆G}_{bind}=∆H-T∆S \end{array}$$

The enthalpy change (∆H) was calculated as the sum of the changes in the gas-phase energy (∆E_MM_) and the free energy of solvation (∆G_sol_), averaged over the conformational systematics generated by the MD simulations:

(2) $$\begin{array}{c} ∆H={∆E}_{MM}+{∆G}_{sol} \end{array}$$

∆E_MM_ was estimated using the following formula:

(3) $$\begin{array}{c} {∆E}_{MM}={∆E}_{ele}+{∆E}_{vdW}+{∆E}_{int} \end{array}$$

where ∆E_ele_, ∆E_vdW_ and ∆E_int_ denote the electrostatic energy, the vdW energy and the internal energies corresponding to the bond, angular, and dihedral angular energies, respectively.

∆G_sol_ is used to express the sum of the polar solvation free energy (∆G_pb_) and the nonpolar solvation free energy (∆G_np_).

(4) $$\begin{array}{c} {∆G}_{sol}={∆G}_{pb}+{∆G}_{np} \end{array}$$

∆G_pb_ was determined by solving the linearized Poisson–Boltzmann equation using the PBSA program in the AMBER 22 suite [38].

### 4.5. Markov Model 

Markov models are capable of grouping repeated states in molecular trajectories that exhibit closely related conformations into microstates. Each microstate represents a state within the Markov model’s state space, and transfer probabilities exist between these states, forming a transfer probability matrix. Through the utilization of this transfer probability matrix and flux analysis techniques, it becomes possible to analyze the dynamics among different microstates [62].

#### 4.5.1. K-Means Clustering Algorithm 

The K-means clustering algorithm is a widely used unsupervised learning method in machine learning clustering techniques. Its operational principle can be summarized as follows: Initially, k cluster centers are initialized based on the distances within a given dataset. Each data object is then assigned to the cluster with the closest distance. Subsequently, the mean value of distances within each cluster is computed to determine new cluster centers, which serve as the basis for repeating the clustering steps. This iterative process continues until the cluster centers no longer change, yielding the final clustering results [63,64].

K-means clustering of the run-out finished trajectories was performed using the RMSD of the atoms as a distance metric. The entire process of Markov model computation was performed using the pyemma software package (version number: 2.5.12) [41]. The two most important parameters in the Markov model are the value of k and the lag time. Taking Free-GlucWT as an example, the k-mean clustering of the trajectories was first performed, and the k-value with the highest score (for 20) was selected based on the scoring results with VAMP-2 [65] (Figure S1). Once the optimal k-value was established, we applied the clustering process to group a large number of molecular conformations from the trajectory. Each cluster in the resulting clustering output was referred to as a microstate. Observing Figure S2 to determine the lag time value, it can be seen that the relaxation time scale of the whole system tends to converge when the lag time is 1.2 ns. Therefore, a lag time of 1.2 ns was chosen for this system. Since the step size used to calculate the molecular simulation trajectory is 0.1 ns, the lag time is 12 steps. The Markov model was ck-tested, and the results obtained are shown in Figure S3, which concluded that the constructed Markov model has good Markovian properties and can be used for subsequent studies. The collection of these microstates formed a discrete trajectory, allowing for analysis of thermodynamic and kinetic relationships through the construction of transfer probability matrices. Flux analysis was performed using the transfer probability matrix of the Markov model. The results of the flux analysis are shown in Figure 7 (the highlighted marked region is the main focus of the studied α-helix region). By analyzing the fluxes and their proportions for each path, Table S1 can be obtained.

#### 4.5.2. Determination of Time Lag Time 

The lag time is a crucial parameter in the calculation of Markov models for constructing transfer matrices. It represents the time interval required for each transformation between discrete trajectories and significantly influences the resulting matrix values. In the Markov model operation, discrete trajectories are transformed at specific lag time intervals, and the resulting jump information is recorded in a counting matrix. Subsequently, this counting matrix is converted into a transfer probability matrix. 

The Markov time represents the minimum lag time required for achieving Markovian behavior, which is characterized by memoryless dynamics. The selection of an appropriate lag time can be assessed by plotting the lag time against the implied timescales [66]. 

As the lag time increases, the implied timescales tend to converge (Figure S2). To further validate the suitability of the chosen lag time, the Chapman–Kolmogorov test (ck-test) can be employed for secondary testing (Figure S3) [67]. The relevant equations involved are as follows:

(5) $$\begin{array}{c} P\left(k\tau \right)=P^{k}\left(\tau \right) \end{array}$$

The approach involves evaluating both sides of the equations listed, and if the results are within a 95% agreement, the lag time is generally deemed to be a reasonable choice [68]. In the equations, P denotes the transfer probability matrix, τ represents the lag time, and k signifies any positive integer. The commonly used default value for k is 5.

#### 4.5.3. Flux Analysis 

The objective of flux analysis is to derive the transfer trajectories within the microstate space constructed by the model. To accomplish this, it requires both the probability distribution matrix and the fluxes.

To begin, the forward probability $\;q_{i}^{+}\;$ and the backward probability $q_{i}^{-}$ are computed individually for each stabilized microstate. This calculation is performed as follows (where $\;T_{ij}$ represents the transfer probability between two microstates obtained from the transfer probability matrix, and A and B denote the microstates at the two endpoints, respectively) [69]:

(6) $$\begin{array}{c} q_{i}^{+}=\sum_{j\in B}T_{ij}+\sum_{j\in \bar{A\cup B}}T_{ij}q_{j}^{+} \end{array}$$

(7) $$\begin{array}{c} q_{i}^{-}=1-q_{i}^{+} \end{array}$$

Once $q_{i}^{+}$ and $q_{i}^{-}$ are computed, the effective flux ${\;f}_{ij}\;$ can be calculated to represent the transition from microstate i to microstate j. To determine the net flux from microstate i to microstate j, two effective fluxes, $f_{ij}\;$ and $f_{ji}$ , are utilized. The calculations are performed as follows (where ${\;\rho }_{i}$ represents the Boltzmann probability of state i):

(8) $$\begin{array}{c} f_{ij}={{{\rho_{i}q}_{i}^{-}T}_{ij}q}_{j}^{+} \end{array}$$

(9) $$\begin{array}{c} f_{ij}^{+}=max\left(f_{ij}-f_{ji}\right) \end{array}$$

To analyze trajectories, the underlying dynamic relationships among intermediate states can be examined by specifying the initial microstate A and the final microstate B. By determining the net fluxes of each intermediate state, various possible paths from microstate A to microstate B can be explored. It is important to note that the paths experienced are not limited to a single route. The probability of occurrence for different paths can be calculated as follows:

(10) $$\begin{array}{c} P_{i}^{A\to B}=\frac{f_{i}^{A\to B}}{\sum_{j}f_{j}^{A\to B}} \end{array}$$

Here, P represents the probability of a specific path compared to the total number of path cases. By enumerating and analyzing all possible path cases, we can identify and prioritize the path cases with the highest probability of occurrence.

#### 4.5.4. Neural Relationship Inference 

The NRI model [70] comprises two co-training components: an encoder, which predicts interactions based on a given dynamical system trajectory, and a decoder, which predicts the dynamical system trajectory based on the interaction graph [71,72].

In the context of the NRI model, the input comprises N nodes. Each node, denoted as node i, has a feature vector ${\;x}_{i}^{t}\;$ at time t representing its position and velocity in the x, y, and z dimensions, resulting in a dimensionality of six for each node. The feature set of all N nodes at time t is represented as ${\;x}^{t}=\{x_{1}^{t},\ldots ,x_{N}^{t}\}$ . The trajectory of node i over T time steps is denoted as $\;x_{i}=\{x_{i}^{1},\ldots ,x_{i}^{T}\}$ , where T is the total number of time steps. Ultimately, all trajectory data is recorded as $x=\{x^{1},\ldots ,x^{T}\}$ . The NRI model learns the edge values and reconstructs the future trajectories of the dynamical system simultaneously and in an unsupervised manner, relying on an unknown graph z.

The interactions between nodes i and j take the form of latent variable $\;z_{i,j}\in \{1,\ldots ,K\}$ , where K is the number of interaction types being modeled. These interaction types do not have any predefined meanings; rather, the model learns to assign a meaning to each type.

Jingxuan Zhu et al. adapted the NRI model to understand how the metastructuring pathway mediates the remote regulation from the ligand-binding or mutation site to the active center of the protein [53]. Based on trajectories simulated by MD, Jingxuan Zhu et al. formulated the protein-mutagenesis process as a dynamic network of interacting residues. The model uses a Graph Neural Network (GNN) to learn the embedding of the network dynamics by minimizing the reconstruction error between the reconstructed trajectory and the simulated trajectory; the NRI model then infers the edges between the residues represented by the latent variables. The learned embeddings essentially abstract the important role of key residues in conformational transitions, which helps to decipher the mechanism of protein isomers. We ran the NRI model using the Cα data from finished trajectories to analyze long-range interactions in molecular dynamics simulations using deep learning.

## 5. Conclusions

In this study, 450 ns molecular dynamics simulations were used to investigate six systems: Free-GlucWT, Free-GlucM1, Free-GlucM2, and GlucWT-CTZ, GlucM1-CTZ, GlucM2-CTZ. The results showed that the glow-type mutant GlucM1 formed more hydrogen bonds and shorter bond lengths after binding to the substrate CTZ, and the RMSD, Rg values of the GlucM1-CTZ system were lower than those of the GlucWT-CTZ and the GlucM2-CTZ during 450 ns MD simulations. Furthermore, MM/PBSA showed that the GlucM1-CTZ system had a lower binding free energy. All results indicate that the GlucM1-CTZ system was more stable. Upon incorporation of the substrate CTZ, the α-helix at residues 109–119 of the flash-type mutant GlucM2 partially unraveled, while the α-helix at residues 109–119 of the glow-type mutant GlucM1 partially formed markedly. The results of Markov flux analyses showed that the conformational differences between the glow-type and flash-type mutants upon binding of the substrate CTZ were mainly involved in the change of α-helicity of α7. Both the most representative conformations and the active pocket distances indicated a higher degree of active site closure and tighter binding in the glow-type mutant GlucM1 relative to the flash-type mutant GlucM2. In summary, we provide a theoretical basis for exploring the relationship between the conformational changes of Gluc mutants and their bioluminescent properties, which can provide a reference for the modification and evolution of luciferase.

## Figures and Tables

**Table 1:** The MM/PBSA results (Kcal/mol) for GlucWT-CTZ.

Energy Component	Average	Std. Dev.	Std. Err. of Mean
∆E_vdW_	−65.7423	4.2555	0.2006
∆E_ele_	−101.9711	31.8121	1.4996
∆G_gas_	−167.7133	33.4725	1.5779
∆G_solv_	126.3252	30.6287	1.4438
∆G_total_	−41.3882	5.8702	0.2767

**Table 2:** The MM/PBSA results (Kcal/mol) for GlucM1-CTZ.

Energy Component	Average	Std. Dev.	Std. Err. of Mean
∆E_vdW_	−65.4621	4.5203	0.2131
∆E_ele_	−106.6130	36.7542	1.7326
∆G_gas_	−172.0751	36.7367	1.7318
∆G_solv_	125.5495	31.0207	1.4623
∆G_total_	−46.5255	7.8161	0.3685

**Table 3:** The MM/PBSA results (Kcal/mol) for GlucM2-CTZ.

Energy Component	Average	Std. Dev.	Std. Err. of Mean
∆E_vdW_	−60.0448	5.8718	0.2768
∆E_ele_	−156.8636	30.0942	1.4187
∆G_gas_	−216.9083	31.1263	1.4673
∆G_solv_	179.5908	29.8570	1.4075
∆G_total_	−37.3175	6.0562	0.2855

## Data Availability

Data are contained within the article and Supplementary Materials.

## Supplementary Materials

The following supporting information can be downloaded at: https://www.mdpi.com/article/10.3390/molecules29174029/s1, Figure S1: (a) The K value determination of Free-GlucWT. (b) The K value determination of Free-GlucM1. (c) The K value determination of Free-GlucM2. (d) The K value determination of GlucWT-CTZ. (e) The K value determination of GlucM1-CTZ. (f) The K value determination of GlucM2-CTZ; Figure S2: (a) The lag time determination of Free-GlucWT. (b) The lag time determination of Free-GlucM1. (c) The lag time determination of Free-GlucM2. (d) The lag time determination of GlucWT-CTZ. (e) The lag time determination of GlucM1-CTZ. (f) The lag time determination of GlucM2-CTZ; Figure S3: (a) The results of ck-test of Free-GlucWT. (b) The results of ck-test of Free-GlucM1. (c) The results of ck-test of Free-GlucM2. (d) The results of ck-test of GlucWT-CTZ. (e) The results of ck-test of GlucM1-CTZ. (f) The results of ck-test of GlucM2-CTZ; Figure S4: The Markov protein conformation alignment map of the highest probability path. (a) The alignment map of Free-GlucWT. (b) The alignment map of Free-GlucM1. (c) The alignment map of Free-GlucM2. (d) The alignment map of GlucWT-CTZ. (e) The alignment map of GlucM1-CTZ. (f) The alignment map of GlucM2-CTZ; Figure S5. RMSD values of substrate CTZ in MD simulations; Table S1. The flux analysis data of Free-GlucWT, Free-GlucM1, Free-GlucM2, GlucWT-CTZ, GlucM1-CTZ, and GlucM2-CTZ.
