# Supplementary material for: Molecular Dynamics Simulation Combined with Neural Relationship Inference and Markov Model to Reveal the Relationship between Conformational Regulation and Bioluminescence Properties of Gaussia Luciferase
Source: Molecules. 2024 Aug 26;29(17):4029. doi: 10.3390/molecules29174029 (PMC11396600; doi:10.3390/molecules29174029)
Supplement: Supplementary file 1 [file molecules-29-04029-s001.zip › molecules-3095517-supplementary.pdf]

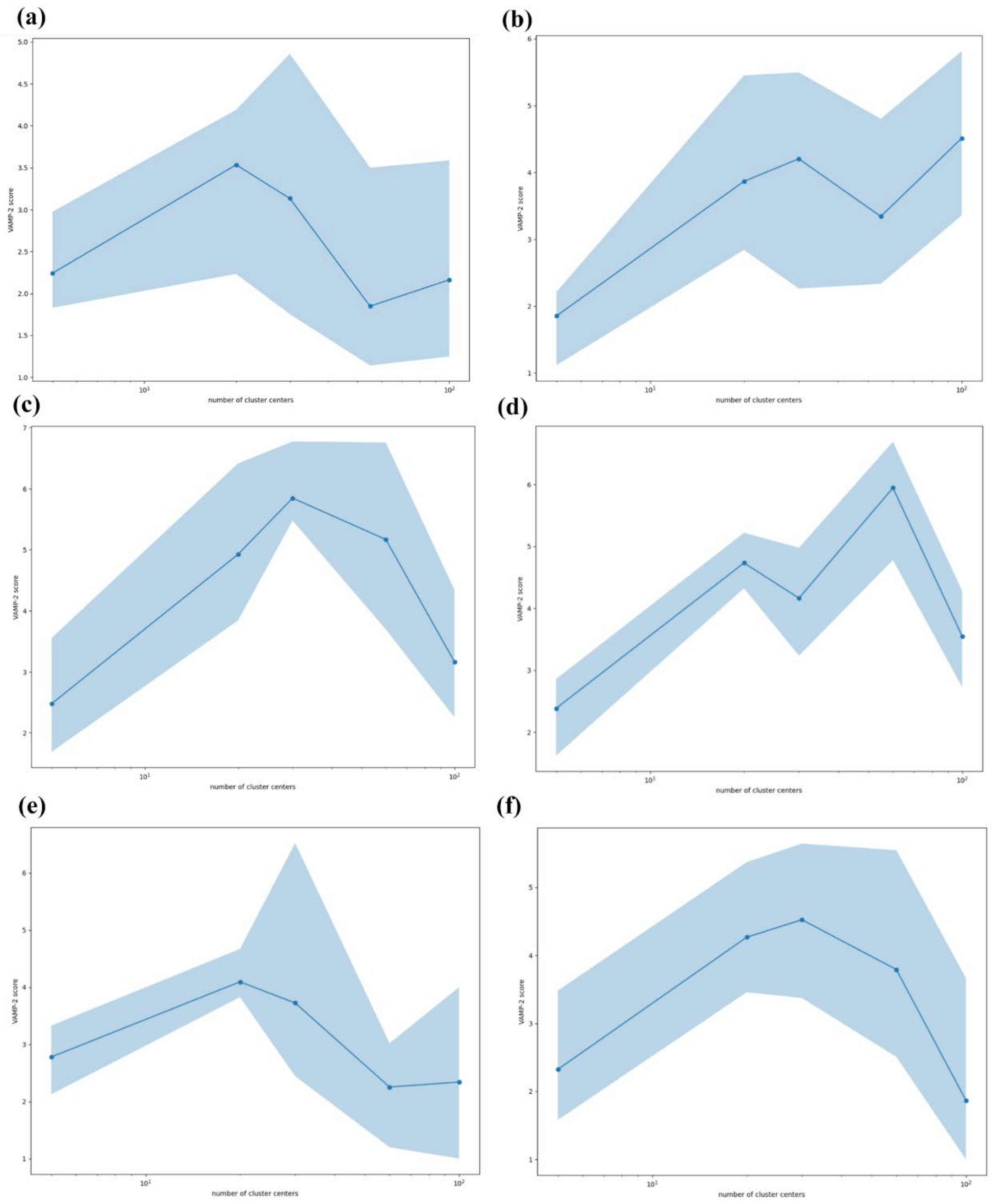

Figure S1. (a) The K value determination of Free-GlucWT. (b) The K value determination of Free-GlucM1. (c) The K value determination of Free-GlucM2. (d) The K value determination of GlucWT-CTZ. (e) The K value determination of GlucM1-CTZ. (f) The K value determination of GlucM2-CTZ.

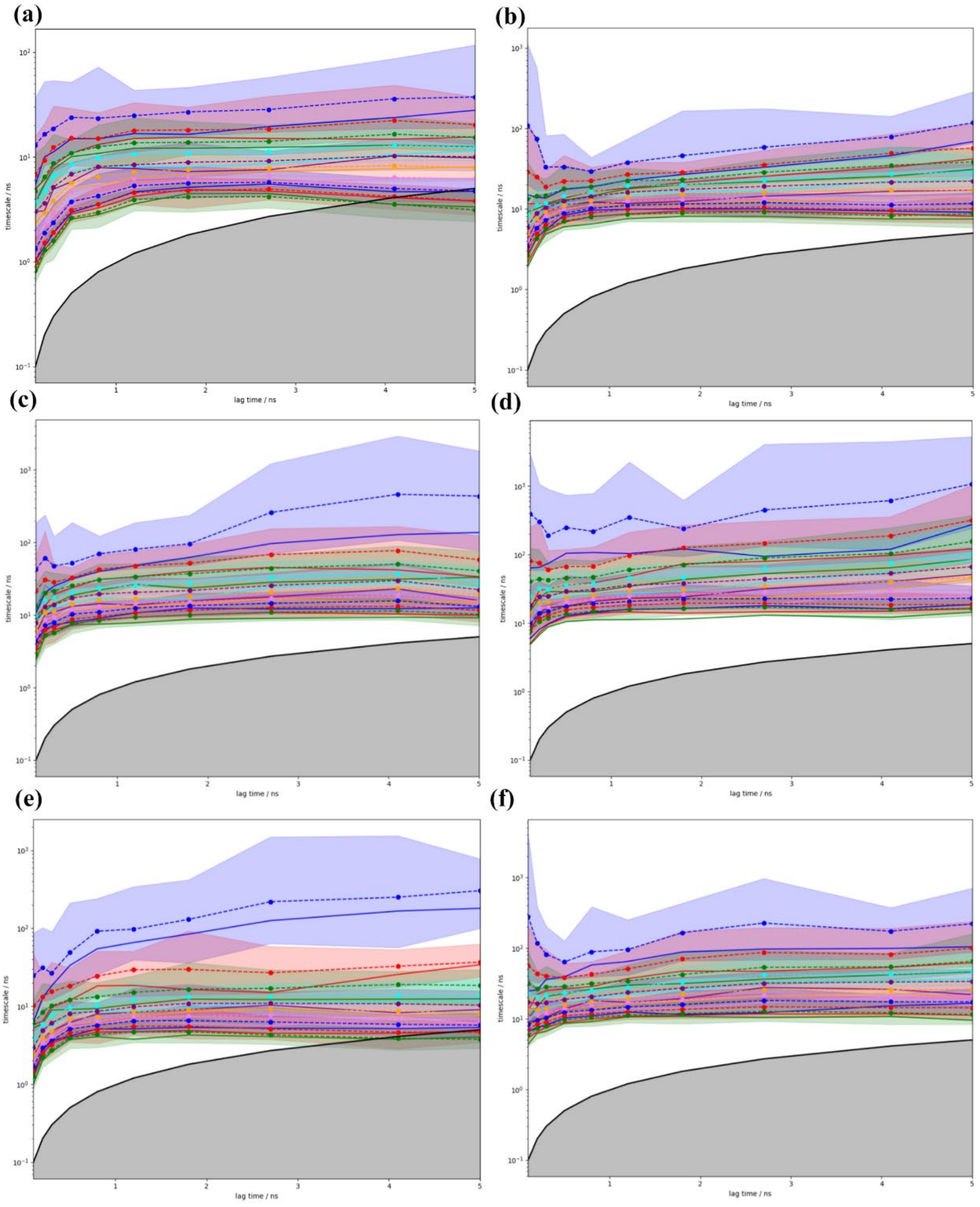

Figure S2. (a) The lag time determination of Free-GlucWT. (b) The lag time determination of Free-GlucM1. (c) The lag time determination of Free-GlucM2. (d) The lag time determination of GlucWT-CTZ. (e) The lag time determination of GlucM1-CTZ. (f) The lag time determination of GlucM2-CTZ.

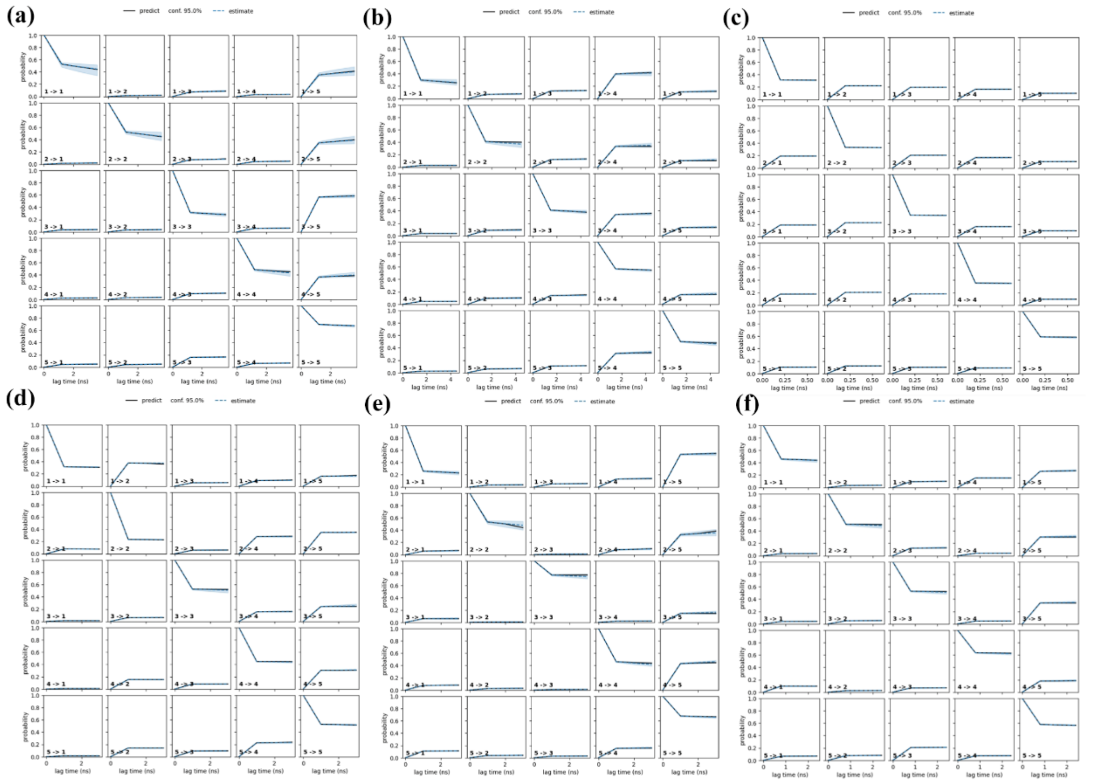

Figure S3. (a) The results of ck-test of Free-GlucWT. (b) The results of ck-test of Free-GlucM1. (c) The results of ck-test of Free-GlucM2. (d) The results of ck-test of GlucWT-CTZ. (e) The results of ck-test of GlucM1-CTZ. (f) The results of ck-test of GlucM2-CTZ.

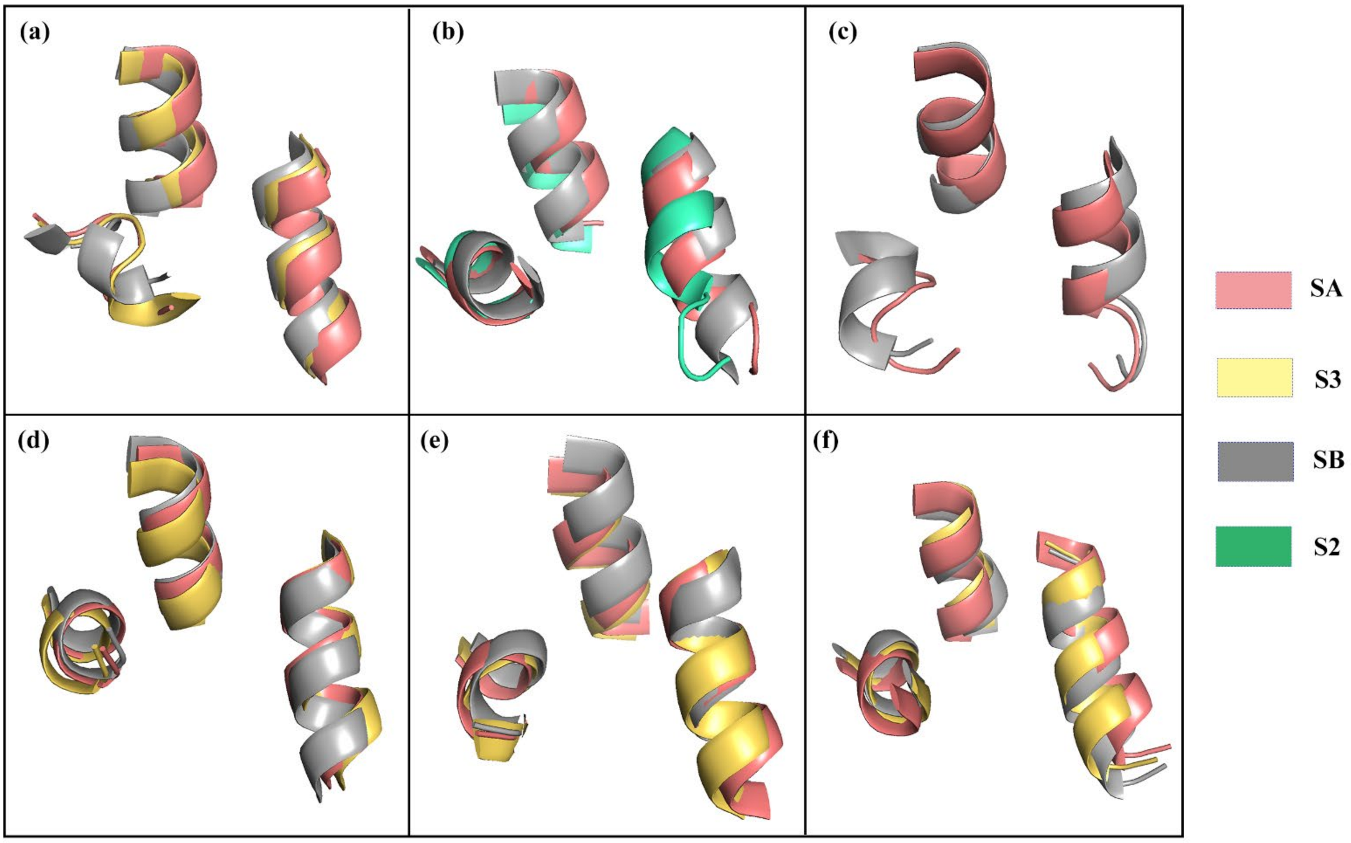

Figure S4. The Markov protein conformation alignment map of the highest probability path. (a) The alignment map of Free-GlucWT. (b) The alignment map of Free-GlucM1. (c) The alignment map of Free-GlucM2. (d) The alignment map of GlucWT-CTZ. (e) The alignment map of GlucM1-CTZ. (f) The alignment map of GlucM2-CTZ.

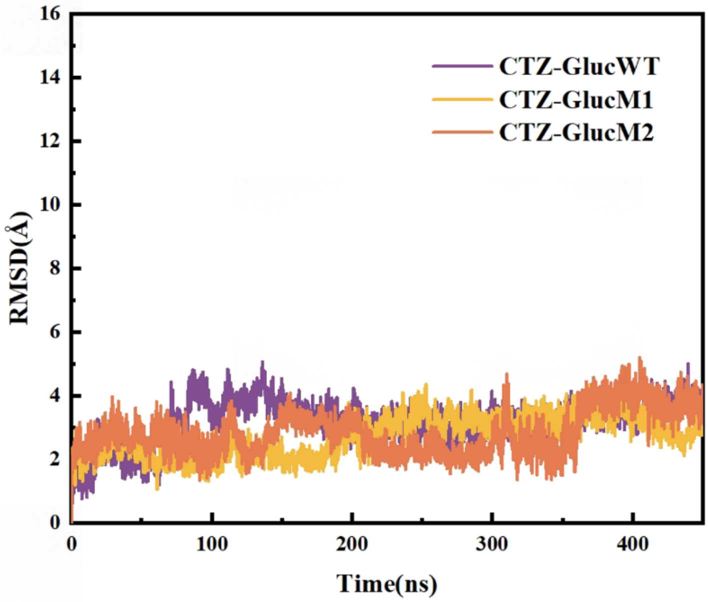

Figure S5. RMSD values of substrate CTZ in MD simulations.

Table S1. The flux analysis data of Free-GlucWT, Free-GlucM1, Free-GlucM2, GlucWT-CTZ, GlucM1-CTZ, and GlucM2-CTZ.

System	Pathways	Path Flux (s^{-1})	Percentage of Total Coarse Flux (%)
Free-GlucWT	SA→S3→SB	2.00×10^{-3}	100.00
Free-GlucM1	SA→S2→SB	6.00×10^{-4}	100.00
Free-GlucM2	SA→SB	5.00×10^{-2}	96.71
	SA→S3→SB	9.00×10^{-4}	1.74
	SA→S2→SB	6.00×10^{-4}	1.16
	SA→S1→SB	2.00×10^{-4}	0.39
	Total	5.17×10^{-2}	100.00
GlucWT-CTZ	SA→S3→SB	1.99×10^{-3}	99.50
	SA→S3→S1→S2→S3→SB	1.00×10^{-5}	0.50
	Total	2.00×10^{-3}	100.00
GlucM1-CTZ	SA→S3→SB	2.00×10^{-3}	100.00
	SA→S3→S2→S1→S3→SB	1.00×10^{-8}	0.00
	Total	2.00×10^{-3}	100.00
GlucM2-CTZ	SA→S3→SB	2.00×10^{-3}	100.00
